# Associations between enamel defects and caries in patients with Down syndrome compared with normotypic patients

**DOI:** 10.1590/1807-3107bor-2025.vol39.075

**Published:** 2025-07-07

**Authors:** Paula Esther Alves CRUZ, Juliane Rolim de LAVÔR, Samylla Glória de Araújo COSTA, Manuella Azevedo Varjal Carneiro LEÃO, Thaysa Gomes Ferreira Tenório dos SANTOS, Aronita ROSENBLATT

**Affiliations:** (a) Universidade de Pernambuco – UPE, School of Dentistry, Department of Pediatric Dentistry, Recife, PE, Brazil.

**Keywords:** Developmental Defects of Enamel, Dental Caries, Down Syndrome

## Abstract

This study aimed to explore the prevalence of developmental defects of enamel (DDE) and dental caries in children with and without Down syndrome (DS) and evaluate potential associations between these conditions. This was a cross-sectional, exploratory, census-based study with children of both sexes aged 6 to 11 years. The sample consisted of 10 children diagnosed with DS and 61 without DS. Standardized forms and records of dmft/DMFT and modified developmental defects of enamel (mDDE) were used. Inter- and intraexaminer agreement was assessed, with an intraclass correlation coefficient (ICC) of 0.93 for DDE and 0.81 for caries. The data were tabulated using SPSS 20.0. Categorical variables were subjected to Pearson’s chi-square test to identify significant associations (p = 0.05). The Kolmogorov–Smirnov test revealed a nonnormal distribution pattern of the data (p <0.001). Therefore, numerical variables were subjected to the Mann–Whitney test to verify statistically significant differences (p = 0.05). Among the 71 study participants, 43 (60.6%) were male, and 28 (39.4%) were female. Among them, 10 (14.1%) had DS, 42 (59.2%) had caries, and 19 (26.8%) had DDE, with demarcated opacity as the most common type of DDE. A significant association was identified between having DS and not having dental caries (p = 0.007) and between having DS and having DDE (p <0.001). Individuals with DS were found to have 9.8 times greater odds of having DDE (p = 0.018). Compared with normotypic children, children with DS had a greater prevalence of DDE and a lower prevalence of dental caries. Additionally, children without DS were more likely to have caries in the presence of DDE.

## Introduction

Down syndrome (DS) is one of the most common congenital anomalies, with an incidence of 1 in 1,000 live births globally.^1^ This genetic condition is caused by the presence of an extra copy of chromosome 21. The condition can occur in three forms: standard trisomy 21, which accounts for approximately 95% of cases and results from an extra copy of chromosome 21 in all cells; translocation, which is present in 3–4% of cases, where the material from chromosome 21 is attached to another chromosome; and mosaicism, which occurs in 1–2% of cases and involves the presence of two or more genetically distinct cell lines within the individual. The latter two forms can result in variation in the presentation of the syndrome’s symptoms.^2,3^ The exact etiology of DS has yet to be established in the literature, but studies point to advanced maternal and/or paternal age as the main determining factor.^4^

The overexpression of certain genes located on chromosome 21 determines the phenotypic characteristics of individuals with DS.^5^ These clinical features include short stature, small and broad hands and feet, a single palmar crease, and a curved fifth finger (clinodactyly).^6,7^In the oral cavity, notable traits such as a smaller maxilla than the mandible, delayed eruption of primary and permanent dentition, tooth agenesis, a high-arched palate, a fissured tongue, macroglossia, a low incidence of caries, and a high prevalence of periodontal disease have been reported.^4^ Despite these distinct phenotypic traits, DS can be definitively diagnosed only through cytogenetic investigation to identify the syndrome’s karyotype, as individuals may exhibit varied characteristics and signs.^8^

Among the oral features of DS, the formation of dental enamel is particularly important. Amelogenesis is a complex biological process involving the interaction of proteins, proteinases, and minerals to form enamel, with amelogenin (encoded by the AMELX gene) being the most abundant protein.^9^ This protein, secreted by ameloblasts, plays a crucial role in the development of the enamel matrix.^10^ Variations in the AMELX gene, as discussed by Tremillo-Maldonado et al.,^11^ can lead to altered amelogenin function, potentially contributing to the formation of developmental defects of enamel (DDE). These defects may further interact with other phenotypic traits associated with DS, influencing the overall oral health of affected individuals.

Depending on the stage of odontogenesis in which ameloblasts are affected, different types of developmental defects can arise, impacting both the quantity and quality of enamel.^12,13^ Enamel hypoplasia occurs when disturbances take place during the embryonic phase of enamel secretion, resulting in reduced thickness and an irregular structure. In contrast, disturbances during the maturation phase lead to hypomineralized enamel, which is characterized by a smooth surface with altered translucency and is clinically classified as either demarcated or diffuse opacity.^12-14^ These defects not only compromise enamel integrity but might also influence susceptibility to oral conditions such as dental caries.

Dental caries, a multifactorial disease, is shaped by a complex interplay of determinants, including socioeconomic, demographic, and cultural factors, as well as biological conditions and habits.^15^ Among these factors, DDE has been identified as a biological risk factor for the development of caries.^13,15^ However, studies exploring the association between DDE and dental caries in individuals with DS are scarce. This study represents an initial exploration of the prevalence of DDE and dental caries in children with and without Down syndrome. By evaluating these conditions in a population from Recife, Brazil, this study aims to address an important gap in the literature and provide insights into potential associations between DDE and dental caries, contributing to a better understanding of oral health in these groups.

## Methods

This research followed the guidelines of Resolution 466/2012 of the Brazilian National Health Council and was submitted to the Research Ethics Committee of the University of Pernambuco under CAAE: 68687223.1.0000.5207 and approval number: 6.049.715. Only children whose guardians signed the informed consent form and the informed assent form participated in this study.

The census sample consisted of ten children with Down syndrome and 61 normotypic children. All children with DS were patients at the Novo Rumo Association, an institution funded by civil society donations that offers multidisciplinary professional assistance to babies, children, and adolescents with developmental delays, located in Casa Amarela, Recife, PE. The normotypic children were enrolled at Luiz Lua Gonzaga School, which is affiliated with Sanitary District II of Recife’s municipal government and located in Bomba do Hemetério, Recife, PE. A census was conducted at both institutions, and all children aged 6 to 11 years of both sexes who were present during the data collection period were assessed. However, only those meeting the eligibility criteria were included in the study. Children who underwent orthodontic treatment with fixed appliances or crowns, and/or children who presented with dental anomalies such as microdontia, macrodontia, talon cusps, and enamel pearls, among other anomalies, were excluded from the study, as were those with systemic and/or mental diseases that limited their participation.

Considering the lack of previous studies addressing the prevalence of DDE in individuals with Down syndrome, this research was designed as an exploratory, census-based study. While the sample size of individuals with Down syndrome is smaller than ideal for achieving high statistical power, the inclusion of all eligible individuals during the data collection period ensures that the findings are representative of the accessible population.

A team of undergraduate students trained by dentists, along with doctoral students from the University of Pernambuco, was formed to collect data. These students were properly trained by pediatric dentistry professors to ensure accuracy in diagnosing caries and developmental defects of enamel.

The team’s training involved studying the theoretical criteria for diagnosing DDE and caries, followed by practical training using photographs. A pilot study was conducted with 20 photographs to identify potential errors and to test and validate the proposed method. Three weeks later, the first evaluation was conducted, incorporating all necessary adjustments identified in the pilot. Inter- and intraexaminer agreement was assessed by a second evaluation of the images after three weeks.

### Data Collection

The participants were examined in a knee-to-knee position at the institutions and in well-lit and appropriate locations for conducting a clinical visual examination using a wooden spatula, diagnostic explorer probes and gauze. Standardized forms were used for patient identification and for recording the decayed, missing, and filled primary teeth (dmft) and decayed, missing, and filled permanent teeth (DMFT) indices,^16,17^ as well as the modified DDE (mDDE) index.^18^

The dmft/DMFT indices were used to quantify the average number of decayed, missing, and filled teeth. The mDDE index was classified as ‘normal’ (Code 0), ‘demarcated opacity’ (Code 1), ‘diffuse opacity’ (Code 2), ‘hypoplasia’ (Code 3), and ‘other defects’ (Code 4). Combinations of defects were classified as ‘demarcated and diffuse’ (Code 5), ‘demarcated and hypoplasia’ (Code 6), ‘diffuse and hypoplasia’ (Code 7), and ‘all three defects’ (Code 8). Additionally, Code 9 corresponded to ‘unable to observe.’

### Statistical Analysis

The data were tabulated using SPSS 20.0 statistical software. Descriptive statistical analysis was used to identify the absolute and relative frequencies of categorical variables, as well as the means, standard deviations, minimums, and maximums of numerical variables. Categorical variables were subjected to Pearson’s chi-square test to identify significant associations (p = 0.05). The Kolmogorov–Smirnov test revealed a nonnormal distribution pattern of the data (p < 0.001). Numerical variables were subjected to the Mann–Whitney test to verify statistically significant differences (p = 0.05). To address potential limitations associated with the small sample size of individuals with Down syndrome, logistic regression was performed, adjusting for confounding factors such as age and sex.

## Results

Inter- and intraexaminer agreement, assessed through the training process using photographic images, demonstrated excellent reproducibility. A second evaluation of the images, conducted three weeks after the initial analysis, resulted in an intraclass correlation coefficient (ICC) of 0.93 for DDE and 0.81 for caries. According to the established criteria, an ICC ≥ 0.75 confirmed the high reliability of the measurements.

The final study sample consisted of 71 children, 43 (60.6%) of whom were male and 28 (39.4%) of whom were female. There were 10 (14.1%) individuals with Down syndrome, 42 (59.2%) with dental caries, and 19 (26.8%) with DDE, and the average age was 7.27 years. Among those examined, demarcated opacity was the most common type of DDE. The average dmft index was 1.87 (±2.255), a standard deviation that reflects the concentration of caries in a few children, and the DMFT index was 0.13 (±0.412). These data are presented in Tables 1 and 2.

In the bivariate analysis using Pearson’s chi-square test, a statistically significant association was identified between having Down syndrome and not having dental caries (p = 0.007) as well as between having Down syndrome and DDE (p < 0.001) (Table 3).

**Table 3:** Bivariate analysis of the presence of dental caries and DDE in the sample (n = 71).

Variables	DS				p- value
	Absent		Present		
	n	%	n	%	
Dental caries					
Absent	21	34,4	8	80	0.007*
Present	40	65,6	2	20	
DDE					
Absent	50	82	2	20	< 0.001*
Present	11	18	8	80	
Total	61	85,9	10	14,1	

DS: Down syndrome; DDE: developmental defects of enamel. *Pearson’s chi-square test.

In the multivariate analysis adjusted for the confounding factors of sex and age (Table 4), the binary logistic regression model revealed that individuals with Down syndrome were 9.8 times more likely to have DDE, a finding that was statistically significant (p = 0.018).

**Table 4:** Univariate and multivariate analyses of the association between Down syndrome and the presence of dental caries and Developmental Defects of Enamel (DDE) In the sample (n = 71).

Variabes	DS Present			
	Crude OR (95%CI)	p-value	Adjusted OR* (95%CI)	p-value**
Dental caries				
Absent	1	-	1	-
Present	0,1 (0,0–0,6)	0.015	0,2 (0,0–1,4)	0.130
DDE				
Absent	1	-	1	-
Present	18,1 (3,3–97,6)	0.001	9,8 (1,4–65,5)	0.018

DS: Down syndrome; OR: odds ratio; DDE: developmental defects of enamel. *Adjusted for sex and age; **Binary logistic regression.

In the analysis of the mean dmft and DMFT indices, as well as their individual components, a significantly greater number of filled primary teeth was observed in individuals with Down syndrome than in those without the condition (Table 5).

**Table 5:** Comparative analysis of the DMFT and dmft indices in the sample (n = 71).

Group	Average	SD	Median	p-value
DMFT				
Non-DS	0.15	0.441	0.00	0.263
DS	0.00	0.000	0.00	
dmft				
Non-DS	2.00	2,191	2.00	0.068
DS	1.10	2,601	0.00	
Number of decayed primary teeth				
Non-DS	1.67	1,895	1.00	0.068
DS	1.00	2,539	0.00	
Number of filled primary teeth				
Non-DS	0.00	0.000	0.00	0.014
DS	0.10	0.316	0.00	
Number of missing primary teeth				
Non-DS	0.36	0.797	0.00	0.095
DS	0.00	0.000	0.00	
Number of decayed permanent teeth				
Non-DS	0.20	0.511	0.00	0.198
DS	0.00	0.000	0.00	
Number of filled permanent teeth				
Non-DS	0.15	0.401	0.00	0.228
DS	0.00	0.000	0.00	
Number of missing permanent teeth				
Non-DS	0.03	0.256	0.00	0.686
DS	0.00	0.000	0.00	

The associations between DDE and dental caries were analyzed in children with and without Down syndrome (Table 6). A chi-square test revealed a significant difference between the groups (p = 0.0002), with children without Down syndrome presenting higher odds of dental caries in the presence of DDE.

**Table 6:** Comparison of dental caries and developmental defects of enamel (DDE) between children with and without Down syndrome.

Variables	DS	Non-DS	OR (95%CI)	p-value*
Dental caries present	2	40	14.54	0.0002
DDE present	8	11	(2.69–78.59)	

*Pearson’s chi-square test.

## Discussion

This study collected data on the association of developmental defects of enamel and dental caries in patients with Down syndrome, compared with normotypic individuals, within a cross-sectional framework. While most research on this topic has focused on nonsyndromic children, few Brazilian studies have addressed the prevalence of these conditions in syndromic patients,^19,20^ highlighting the need to address this gap in the literature.

The literature suggests that there are determining factors in the development of enamel defects, such as environmental and genetic factors.^21^ Events during pregnancy, such as gestational diabetes, hypertension, neonatal hypoxia, and preterm birth, can interfere with mesoblast activity and enamel formation.^22,23^ Postnatal factors, including insufficient breastfeeding, chronic diseases in infancy, and high fever, have also been identified as risk factors for DDE.^21,23^ These environmental influences may exacerbate the genetic predispositions observed in patients with Down syndrome, contributing to the high prevalence of DDE (80%) found in our study population.

The literature also highlights the impact of genetic factors on the development of DDE. According to Li et al.,^24^ overexpression of the RCAN1 gene on chromosome 21 in patients with Down syndrome impairs ameloblast function, leads to mitochondrial dysfunction, and increases oxidative stress levels, all of which contribute to defective enamel. Keinan et al.^25^ further demonstrated that individuals with Down syndrome have altered hydroxyapatite crystal morphology in their enamel, reducing its mechanical properties and acid resistance. These structural differences provide a biological basis for the higher prevalence of DDE observed in our study, which revealed that patients with Down syndrome were 9.8 times more likely to have DDE than nonsyndromic patients were, highlighting the implications of DS for oral health.

Evidence has demonstrated a correlation between enamel hypoplasia and dental caries, with individuals predisposed to conditions such as premature birth or cleft lip and palate being at greater risk of developing caries due to hypoplasia.^13,26^ As a result of its increased porosity and irregular surface, defective dental enamel allows for greater biofilm accumulation, and the sensitivity associated with these defects often impairs proper brushing, making teeth more prone to plaque and, consequently, caries.^13,26^The high prevalence of DDE identified in the present study supports other studies that recognized hypoplasia and hypomineralization as common defects in the teeth of patients with Down syndrome.^27,28^ Since this syndrome affects overall development, alterations during the embryonic phase may lead to changes in both deciduous and permanent dentition.^27^

Although defective enamel is more vulnerable to caries and patients with DS have a higher incidence of DDE, some studies have indicated that these patients are less susceptible to carious lesions.^20,28^ A systematic review by Moreira et al.^29^ similarly reported a lower prevalence of caries in patients with DS, which aligns with the findings of this study, in which only 20% of children with DS had caries, whereas 65.6% of those without DS had caries. Delayed tooth eruption in syndromic patients likely contributes to these findings, as it results in a shorter period of exposure to cariogenic factors than normotypic patients experience. The fact that syndromic patients have a more alkaline saliva pH favors buffering capacity, protecting enamel against acid attacks produced by acidogenic bacteria circulating in the oral environment. Additionally, dental anomalies often present in patients with DS, such as microdontia, can produce diastemas that reduce plaque accumulation on the interproximal surfaces because they facilitate oral hygiene in the area.^20,28,29^ Furthermore, patients with DS are frequently diagnosed with bruxism, which is characterized by flat occlusal surfaces that favor self-cleaning and prevent the development of fissure and pit caries.^28,29^

The prevalence of DDE in the study population without Down syndrome was 18%. Alshehhi et al.^12^reported a similar prevalence of 24.2%. Massignan and collaborators^30^ found a significantly greater prevalence of DDE (39.1%) among children. This difference may be explained by the variation in population age across the different investigations. According to Verma et al.,^23^ DDE in older patients may be masked by the presence of caries, restoration, or tooth wear. Additionally, a global comparative prevalence study showed that DDE rates in deciduous dentition vary between 10% and 49%.^13^

With respect to the prevalence of each type of defect, demarcated opacity was the most prevalent (88.7%), followed by hypoplasia (11.2%), and finally, demarcated opacity associated with hypoplasia (4.2%). Several studies have also reported demarcated opacity as the predominant enamel defect in their samples.^12,19,23,31^

Caries in school-aged children is a global-scale problem. Epidemiological research from different countries revealed prevalence rates varying between 41% and 81% in children aged 6 to 12 years.^32^ In this analysis, a caries prevalence of 65.6% was reported in normotypic children, constituting a concerning rate for our population. The mean DMFT value (0.15) among normotypic participants was classified as very low according to the World Health Organization’s classification.^33^ In contrast, the dmft index was classified as low, with a score of 2.0. This difference between the DMFT and dmft values can be attributed to the age of the sample population, as deciduous teeth tend to remain in the oral cavity longer, whereas permanent teeth are just beginning to erupt at this stage.^32,34^

The limitations of this study arise from the small sample size of patients with Down syndrome, reflecting the reality that these individuals are no longer the primary clientele of institutions for people with special needs in the city of Recife. Additionally, challenges associated with intraoral examinations in syndromic patients, particularly due to cognitive limitations that reduce their ability to cooperate,^20^ also impact data collection. Despite these constraints, the statistical methods employed, including logistic regression adjusted for confounding factors, address some of the limitations associated with the small sample size. While the Down syndrome group is limited in size, this study provides crucial first insights into an underserved population, describing the higher prevalence of DDE found in this population and paving the way for future research. Larger sample sizes and cohorts are needed to confirm these initial observations.

## Conclusion

Compared with normotypic children, children with Down syndrome had a greater prevalence of DDE, with demarcated opacity being the most common defect, and a lower prevalence of dental caries. Additionally, children without Down syndrome were more likely to have caries in the presence of DDE. These findings enhance our understanding of the distinct oral health characteristics of these groups and provide a solid foundation for future research.

**Table 1:** Frequency distribution of Down syndrome, dental caries, and Developmental Defects of Enamel (DDE) among study participants (n = 71).

Variable	n	%
Sex		
Female	28	39.4
Male	43	60.6
Down Syndrome (DS)		
Absent	61	85.9
Present	10	14.1
Dental Caries (per affected individual)		
Absent	29	40.8
Present	42	59.2
DDE (per affected individual)		
Absent	52	73.2
Present	19	26.8
DDE classification (per affected tooth)		
Demarcated opacity	63	88.7
Hypoplasia	8	11.2
Demarcated opacity and hypoplasia	3	4.2
Diffuse opacity	0	0.0

**Table 2:** Descriptive statistics of children’s ages and dental caries indices (dmft/DMFT).

Variable	Mean	Min	Max	SD
Age (in years)	7.27	6	11	1,158
dmft score (per individual)	1.87	0	8	2,255
Decayed (per tooth)	1.58	0	8	1,99
Filled (per tooth)	0.01	0	1	0,119
Missing (per tooth)	0.31	0	4	0,748
DMFT score (per individual)	0.13	0	2	0,412
Decayed (per tooth)	0.54	0	8	1,382
Filled (per tooth)	0.13	0	2	0,375
Missing (per tooth)	0.03	0	2	0,237

## Data Availability

The contents underlying the research text are contained in the manuscript.
